# Apoptotic Volume Decrease (AVD) Is Independent of Mitochondrial Dysfunction and Initiator Caspase Activation

**DOI:** 10.3390/cells1041156

**Published:** 2012-12-05

**Authors:** Emi Maeno, Takeshi Tsubata, Yasunobu Okada

**Affiliations:** 1 Department of Cell Physiology, National Institute for Physiological Sciences, Okazaki 444-8585, Japan; E-Mail: maenoe@hotmail.com; 2 Laboratory of Immunology, Tokyo Medical and Dental University, Graduate School of Biomedical Science, Tokyo 113-8510, Japan; E-Mail: tsubata.imm@mri.tmd.ac.jp

**Keywords:** apoptosis, AVD, mitochondria, caspase-3, caspase-8, caspase-9

## Abstract

Persistent cell shrinkage is a major hallmark of apoptotic cell death. The early-phase shrinkage, which starts within 30−120 min after apoptotic stimulation and is called apoptotic volume decrease (AVD), is known to be accomplished by activation of K^+^ channels and volume-sensitive outwardly rectifying (VSOR) Cl^−^ channels in a manner independent of caspase-3 activation. However, it is controversial whether AVD depends on apoptotic dysfunction of mitochondria and activation of initiator caspases. Here, we observed that AVD is induced not only by a mitochondrial apoptosis inducer, staurosporine (STS), in mouse B lymphoma WEHI-231 cells, but also by ligation of the death receptor Fas in human B lymphoblastoid SKW6.4 cells, which undergo Fas-mediated apoptosis without involving mitochondria. Overexpression of Bcl-2 failed to inhibit the STS-induced AVD in WEHI-231 cells. These results indicate that AVD does not require the mitochondrial pathway of apoptosis. In human epithelial HeLa cells stimulated with anti-Fas antibody or STS, the AVD induction was found to precede activation of caspase-8 and caspase-9 and to be resistant to pan-caspase blockers. Thus, it is concluded that the AVD induction is an early event independent of the mitochondrial apoptotic signaling pathway and initiator caspase activation.

## Abbreviations

AMC: fluorochrome 7-amino 4-methyl coumarin
AVD: apoptotic volume decrease
DIDS: 4,4'-diisothiocyanostilbene-2,2'-disulfonic acid
DMSO: dimethylsulfoxide
FBS: fetal bovine serum
MTT: 3-[4,5-dimethylthiazol-2-yl]-2,5-diphenyltetrazolium bromide
NPPB: 5-nitro-2-(3-phenylpropylamino)benzoic acid
ROS: reactive oxygen species
SITS: 4-acetamido-4'-isothiocyanostilbene
STS: staurosporine
VDAC: voltage-dependent anion channel
VSOR: volume-sensitive outwardly rectifying
zD-dcb: Z-Asp-2,6-dichlorobenzoyloxymethylketone
zVAD-fmk: benzyloxycarbonyl-Val-Ala-Asp(OMe)-fluoromethylketone

## 1. Introduction

A major hallmark of apoptotic cell death is normotonic persistent shrinkage of cells [1]. The early-phase apoptotic whole-cell shrinkage, termed apoptotic volume decrease (AVD) [2,3], starts within 0.5−2 h after apoptosis induction, regardless of whether caspases, central executers of apoptosis, are activated through mitochondrial dysfunction (intrinsic pathway) or directly by death receptor ligation (extrinsic pathway) [2]. A sizable AVD event was recently found to start as early as 2–5 min after stimulation with an intrinsic apoptosis inducer, staurosporine (STS), and to precede activation of JNK and p38 MAPK [4]. AVD is mainly accomplished by KCl release mediated by activation of K^+^ and Cl^−^ channels in a wide variety of cell types [2,3,4,5,6,7,8,9]. The Cl^−^ channel involved in the AVD induction was specified to be the volume-sensitive outwardly rectifying (VSOR) anion channel, which is activated by reactive oxygen species (ROS) [10]. Late-phase apoptotic cell shrinkage observed at around 1 to 12 h after apoptotic stimulation was reported to be preceded by alterations in mitochondrial functions, including changes in the mitochondrial membrane potential and generation of mitochondrial ROS [11,12]. However, it is not known whether the early-phase apoptotic shrinkage, that is AVD, is also dependent on the mitochondrial apoptotic signaling pathway. Thus, the first aim of the present study is to answer this question. 

Apoptotic cell shrinkage is considered to be largely independent of activation of effector caspase-3, because this event was observed even in the presence of the specific caspase-3 inhibitor Asp-Glu-Val-Asp-O-methyl-fluoromethylketone (DEVD-fmk) [13,14]. Also, the AVD induction was found to precede activation of caspase-3 [2]. On the other hand, a pan-caspase inhibitor, benzyloxycarbonyl-Val-Ala-Asp(OMe)-fluoromethylketone (zVAD-fmk), has been reported to suppress apoptotic cell shrinkage observed 1.5−48 h after application of apoptosis inducers in a variety of cell types [13,14,15,16,17,18,19,20,21], suggesting that induction of the late-phase apoptotic shrinkage, at least in part, involves activation of some caspase(s) other than caspase-3, especially initiator caspases that activate caspase-3. In fact, the initiator caspases, caspase-8 and caspase-9, were found to be necessary for Fas- and UV-induced apoptotic cell shrinkage observed at ≥2 h after stimulation in Jurkat cells [20]. However, it is not known whether the earlier caspase-3-independent AVD event is dependent on activation of initiator caspases. The second aim of the present study is to address this question.

## 2. Results

### 2.1. The AVD Induction Is Independent of the Mitochondrial Apoptosis Pathway

**Figure 1:**
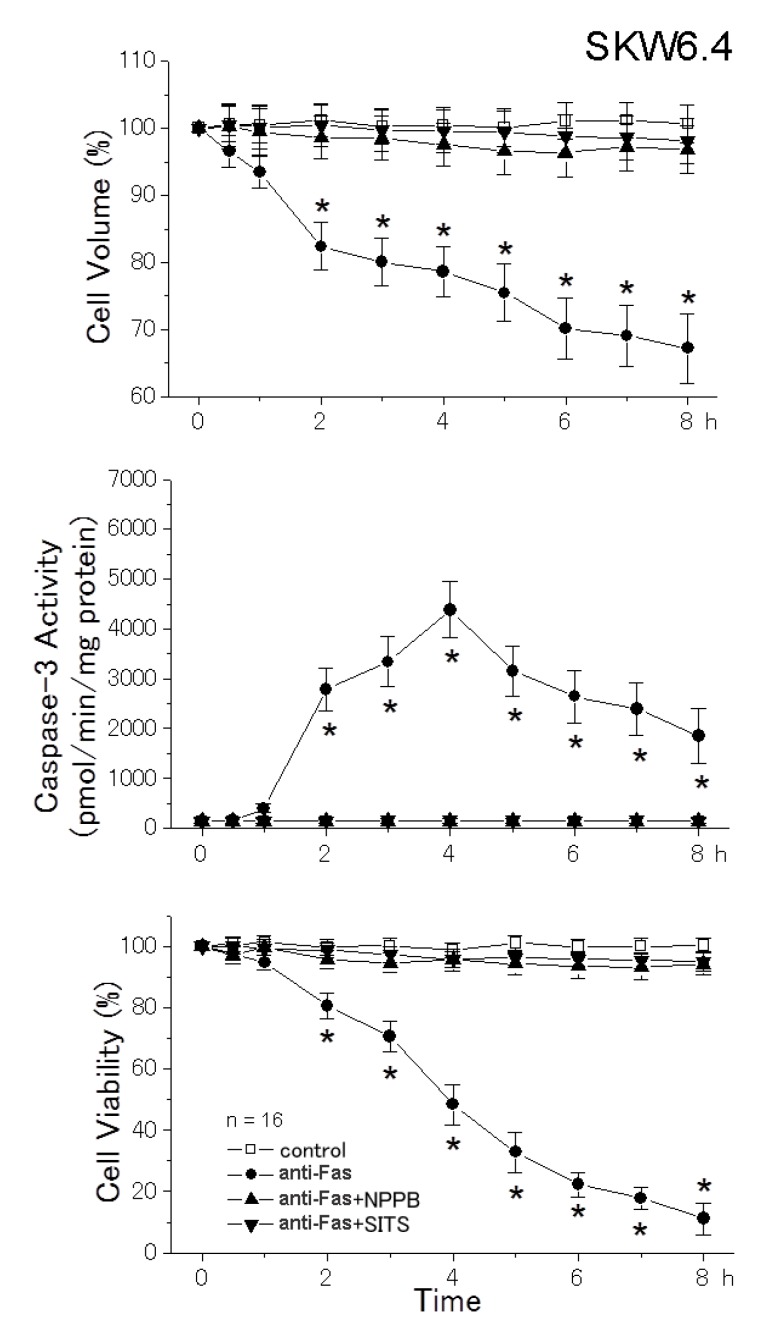
Induction of AVD followed by persistent shrinkage (top), activation of caspase-3 (middle) and induction of cell death (bottom) by treatment with anti-Fas antibody (anti-Fas), as well as their prevention by simultaneous treatment with a Cl^−^ channel blocker (0.5 mM NPPB or 0.5 mM SITS) in SKW6.4 cells. The mean data for cell volume and viability obtained in anti-Fas antibody-treated cells were normalized to those measured in non-treated cells (control). Each data point represents the mean ± SEM (vertical bar) of 16 observations. *p < 0.05 *vs.* corresponding control.

A human B lymphoblastoid cell line SKW6.4 is classified as the type I cell in which death receptor ligation induces apoptosis through the extrinsic pathway [22], because ligation of the death receptor Fas induces apoptosis without mitochondrial apoptotic events in these cells [23]. As shown in Figure 1, treatment of SKW6.4 cells with anti-Fas monoclonal antibody led to not only activation of caspase-3 (middle panel) and reduction of cell viability (bottom panel), but also to induction of AVD, which started before activation of caspase-3, followed by sustained cell shrinkage (top panel) in a time-dependent manner. Thus, it appears that AVD can be induced by the extrinsic apoptosis pathway without involving mitochondria. When a Cl^−^ channel blocker, 5-nitro-2-(3-phenylpropylamino)benzoic acid (NPPB) or 4-acetamido-4'-isothiocyanostilbene (SITS), was simultaneously applied with anti-Fas antibody, AVD, sustained cell shrinkage, caspase-3 activation and cell death were all completely prevented (Figure 1). These results suggest that the mechanism of AVD induction is independent of the mitochondrial pathway and that actions of the Cl^−^ channel blocker were not mediated by the mitochondrial voltage-dependent anion channel (VDAC).

**Figure 2:**
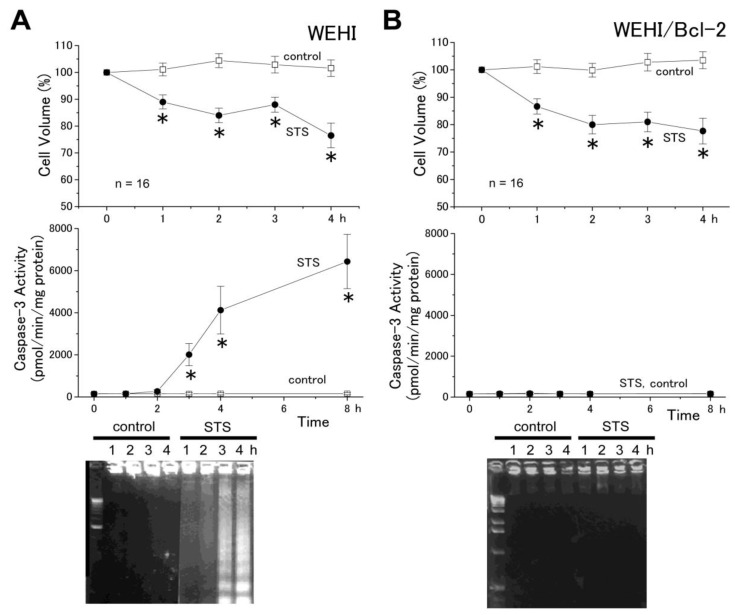
Induction of AVD (top) in both WEHI-231 (**A**) and WEHI-231/Bcl-2 (**B**) cells, as well as activation of caspase-3 (middle) and induction of DNA fragmentation (bottom) in WEHI-231 (A), but not in WEHI-231/Bcl-2 (B) cells treated with STS. The mean cell volume of STS-treated cells was normalized to that of non-treated (control) cells. Each data point represents the mean ± SEM (vertical bar) of 16 observations. *p < 0.05 *vs.* corresponding control.

As shown in Figure 2A, STS also induced AVD (within 1 h), followed by sustained shrinkage (top panel), as well as caspase-3 activation (after ≥2 h: middle panel) and DNA fragmentation (after ≥3 h: bottom panel) in a mouse B lymphoma cell line WEHI-231, in good agreement with previous observations in other cell lines stimulated with STS [2]. Although STS-induced AVD occurred earlier than caspase-3 activation and DNA fragmentation, there remains a possibility that some mitochondrial signal was involved in triggering the AVD induction in this cell line stimulated with the mitochondrion-mediated apoptosis inducer. However, as shown in Figure 2B, the AVD event induced by STS was not prevented by overexpression of Bcl-2 (top panel), which blocks the mitochondrial apoptosis pathway [24] in WEHI-231 cells, whereas caspase-3 activation (middle panel) and DNA fragmentation (bottom panel) were abolished by Bcl-2. These results suggest that the STS-induced process of AVD induction does not require the mitochondrial signaling pathway, which eventually leads to caspase-3 activation and DNA laddering. Taken together, it is concluded that the AVD induction is independent of the mitochondrial pathway leading to apoptotic death in B cells.

### 2.2. The AVD Induction Is an Independent Event of Activation of Initiator Caspases

**Figure 3:**
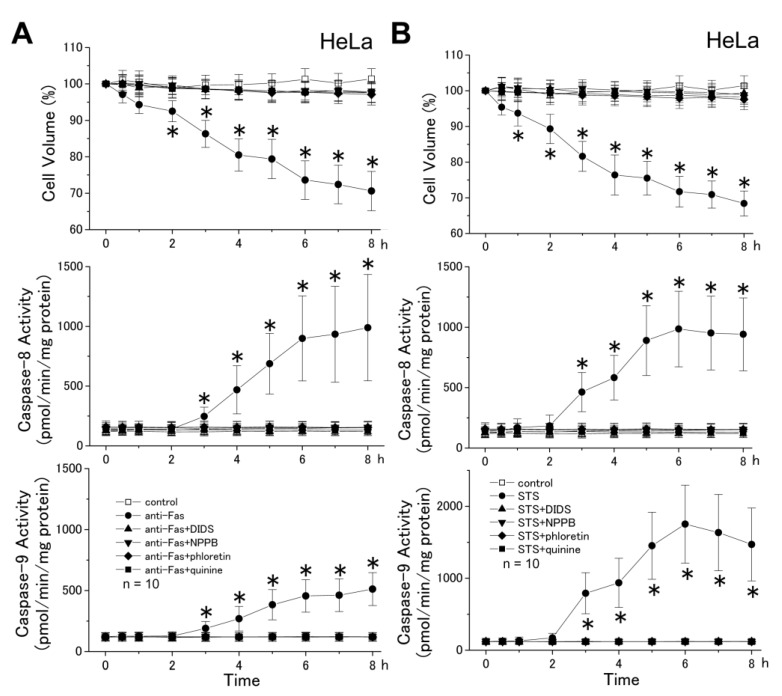
Induction of AVD followed by persistent shrinkage (top), activation of caspase-8 (middle) and activation of caspase-9 (bottom) in HeLa cells treated with anti-Fas antibody (anti-Fas) (**A**) and STS (**B**) in the absence (filled circles), but not in the presence (other filled symbols), of a channel blocker. The Cl^−^ channel blocker was 0.5 mM DIDS, 0.5 mM NPPB, or 30 μM phloretin, and the K^+^ channel blocker was 0.5 mM quinine. The mean cell volume of anti-Fas- and STS-treated cells was normalized to that of non-treated (control: open squares) cells. Each data point represents the mean ± SEM (vertical bar) of 10 observations. *p < 0.05 *vs.* corresponding control.

In a human epithelial cell line HeLa, as shown in Figure 3A and B, stimulation with either anti-Fas antibody or STS induced cell shrinkage (top panels), as well as activation of both caspase-8 (middle panels) and caspase-9 (bottom panels). STS-induced activation of caspase-9 was more prominent than that induced by anti-Fas antibody, presumably because the extrinsic pathway predominantly mediated Fas-induced apoptosis in HeLa cells. The AVD event started very rapidly and became significant as early as 2 h after application of anti-Fas antibody and 1 h after STS application, whereas activation of both initiator caspases was observed only at ≥3 h after apoptotic stimulation. Moreover, prevention of AVD by a non-specific Cl^−^ channel blocker (4,4'-diisothiocyanostilbene-2,2'-disulfonic acid (DIDS) or NPPB), a VSOR anion channel blocker (phloretin), or a K^+^ channel blocker (quinine) completely abolished anti-Fas antibody- and STS-induced late-phase sustained shrinkage, as well as activation of both initiator caspases. These data indicate that the AVD induction is an early event independent of activation of initiator caspases.

**Figure 4:**
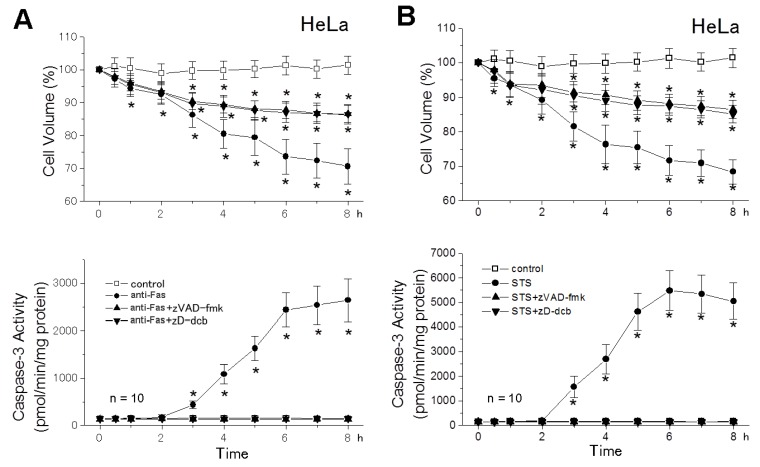
Effects of pan-caspase inhibitors, zVAD-fmk (50 μM) and zD-dcb (100 μM), on the AVD induction (top) and caspase-3 activation (bottom) in HeLa cells treated with anti-Fas antibody (anti-Fas) (**A**) and STS (**B**). The mean cell volume of anti-Fas- or STS-treated cells in the absence (filled circles) and presence (filled triangles) of a pan-caspase blocker was normalized to that of non-treated (control: open squares) cells. Each data point represents the mean ± SEM (vertical bar) of 10 observations. *p < 0.05 *vs.* corresponding control. It is noted that either pan-caspase blocker abolished caspase-3 activation, but not AVD induction in both anti-Fas- and STS-treated cells, and also that the late-phase cell shrinkage observed at ≥4 h after stimulation with anti-Fas or STS was significantly (*p* < 0.05) suppressed by pan-caspase blockers.

A cell-permeable broad-spectrum caspase inhibitor, zVAD-fmk or Z-Asp-2,6-dichlorobenzoyloxymethylketone (zD-dcb), completely abolished activation of caspase-3 (Figure 4: bottom panels) and of caspases-8 and -9 (data not shown, n = 10) upon stimulation with anti-Fas antibody and STS. In contrast, these pan-caspase inhibitors failed to abolish the AVD induced by anti-Fas antibody (Figure 4A: top panel) and STS (Figure 4B: top panel). The AVD occurred within ≥1 h and ≥0.5 h after stimulation with anti-Fas antibody and STS, respectively, and was not affected by the pan-caspase inhibitor up to 2 h after apoptotic stimulation. However, late-phase apoptotic cell shrinkage was largely, though not totally, inhibited by either of pan-caspase inhibitors only at ≥4 h after apoptotic stimulation (Figure 4: top panels), the timing of which matches to that of activation of caspase-3 (Figure 4: bottom panels). These data indicate that the early-phase apoptotic cell shrinkage called AVD does not require activation of caspases including both initiator and effector caspases and that late-phase shrinkage involves both caspase-independent and -dependent mechanisms.

Based on the data shown in Figure 3 and Figure 4, it is concluded that the AVD induction is an independent event of initiator caspase activation.

## 3. Discussion

A reduction in cell volume is a fundamental feature of apoptotic cell death [1]. The apoptotic cell volume loss in CEM-C7A lymphoblastoid cells treated with dexamethasone was characterized by two distinct stages; that is, the first stage (until 36 h) associated with a net loss of K^+^, and the second stage associated with cellular fragmentation (over 36 h) [25]. Here, we found that the first-stage whole-cell shrinkage (before cellular fragmentation or apoptotic body formation) is subdivided into two phases (Figure 4): The early-phase apoptotic shrinkage, AVD, which occurs within 0.5−2 h after apoptotic stimulation in a manner independent of caspase activities and the late-phase persistent shrinkage, which appears at > 2−3 h after apoptotic stimulation in a manner largely dependent on caspase activation. The AVD induction was totally inhibited by blockers of K^+^ and Cl^−^ channels (Figure 1 and Figure 3: top panels), suggesting that electroconductive K^+^ and Cl^−^ efflux is causative of the AVD event. In fact, our recent fluorometric measurements of the intracellular free K^+^ and Cl^−^ concentrations demonstrated early-phase (starting with 10−30 min) occurrence of K^+^ and Cl^−^ effluxes, which are sensitive to a blocker of K^+^ and Cl^−^ channels, respectively [26]. Also, it is noted that abolition of the AVD induction by a blocker of K^+^ or Cl^−^ channel resulted in elimination of the late-phase apoptotic events, including persistent cell shrinkage, caspase-3 activation and cell death (Figure 1), as well as activation of caspases-8 and -9 (Figure 3). Thus, it is suggested that the KCl efflux or resultant AVD is an upstream event of these late-phase apoptotic events.

An extrinsic apoptosis inducer anti-Fas antibody was found to induce the AVD event in the SKW6.4 cell line (Figure 1), which is classified into the type I cell and undergoes Fas-mediated apoptosis without activation of the mitochondrial apoptotic signaling pathway [22]. Also, an intrinsic apoptosis inducer, STS, was found to induce the AVD even in WEHI-231 cells transfected with Bcl-2 (Figure 2B) that protect mitochondria from apoptotic changes. Thus, it appears that the early-phase AVD in apoptotic B cells is independent of the mitochondrial pathway and its downstream event, including caspase-9 activation. Consistently, the AVD induction was found to precede activation of caspase-9 in HeLa cells stimulated with anti-Fas antibody and STS (Figure 3). Also, activation of another initiator caspase, caspase-8, was found to be preceded by the AVD induction in HeLa cells undergoing Fas- and mitochondrion-mediated apoptosis (Figure 3). Moreover, broad-spectrum caspase inhibitors (zVAD-fmk and zD-dcb) failed to affect the apoptotic induction of early-phase (<2 h) whole-cell shrinkage, AVD (Figure 4: top panels). If the caspase assays in the present study were not sensitive enough to detect very small increases in the caspase-8/9 activities, which might have occurred within 0.5−2 h after apoptotic stimulation, there arises a possibility that caspase-8/9 activation in a small extent was involved in the AVD induction. However, this possibility can be excluded by the fact that pan-caspase inhibitors, zVAD-fmk and zD-dcb, which abolished activation not only of caspase-3, but also of caspase-8/9, failed to affect the AVD event up to 2 h after apoptotic stimulation (Figure 4). Therefore, it is evident that neither the mitochondrial apoptotic pathway nor activation of initiator caspases is required for the AVD induction.

The signaling process upstream to AVD is not clear. Since the AVD induction was found to precede activation of JNK and p38 [4], these MAP kinases are unlikely to serve as the upstream signals. There is a possibility that ROS are the upstream signal for AVD, because the VSOR anion channel involved in the AVD induction is known to be activated by ROS [10,27]. In fact, the VSOR channel activation was shown to be mediated by ROS when HeLa cells were stimulated by STS, but not by anti-Fas antibody [10]. However, it must be noted that not only Fas-mediated, but also STS-induced, AVD induction would be independent of mitochondrial ROS production, which is mediated by Romo1 [28] and prevented by Bcl-X_L_ [29], because the present study demonstrated that AVD does not require the mitochondrial pathway (Figure 1) and is insensitive to Bcl-2 (Figure 2). A possible involvement of ROS of non-mitochondrial origin must be examined in the further study.

There has been much discrepancy about the effects of pan-caspase inhibitors on apoptotic cell shrinkage. The pan-caspase inhibitor zVAD-fmk was reported to block apoptotic cell shrinkage in lymphoid cells stimulated with etoposide after 4.5 h [16], glucocorticoids after 4 h [15,17], TGFβ after 48 h [14] and anti-Fas antibody after 1.5−24 h [13,18,19,20], as well as in epidermoid A431 cells at 8 h after UV irradiation [21]. In contrast, the same and several other pan-caspase inhibitors were observed to fail to inhibit apoptotic cell shrinkage in Jurkat cells stimulated with A23187 or thapsigargin after 24 h [13] and with UV irradiation after 8 h [20], as well as in U937 cells stimulated with STS after 2 h [30]. In the present study, the early-phase (until 1−2 h) AVD was found to be totally insensitive to zVAD-fmk and zD-dcb, although the late-phase (over 2−3 h) whole-cell shrinkage was partially suppressed by these pan-caspase inhibitors in HeLa cells stimulated with anti-Fas antibody and STS (Figure 4: top panels). Since late-phase apoptotic shrinkage consisted of caspase-independent and -dependent components, it is likely that the former component represents persistent AVD and that the latter one does additional shrinkage associated with caspase activation. The fact that there are two distinct phases with respect to pan-caspase inhibitor sensitivity may compromise over the discrepancy in the results reported hitherto on sensitivity of apoptotic shrinkage to pan-caspase inhibitors, because a starting time of the late-phase may vary depending on the cell types and apoptotic stimuli.

## 4. Experimental Section

### 4.1. Cell Culture and Apoptosis Induction

HeLa cells were cultured in MEM medium supplemented with 10% fetal bovine serum (FBS). SKW6.4, WEHI-231 and Bcl-2-transfected WEHI-231 (WEHI-231/Bcl-2) cells were cultured in RPMI 1640 medium supplemented with 10% FBS.

To induce apoptosis, these cells in the log-growing phase were treated with 4 μM STS (Sigma, St. Louis, MO, USA) or 0.5 μg/mL anti-Fas monoclonal antibody (MBL, CH-11, Tokyo, Japan), as previously described [2,22,31].

### 4.2. Cell Volume Measurements

Cell volume was measured at room temperature (23−26°C) by applying an electronic cell sizing technique to suspensions of isolated cells, as described previously [26,32]. The standard solution was made of (mM) 95 NaCl, 4.5 KCl, 1 MgCl_2_, 1 CaCl_2_, 5 HEPES/NaOH and 105 mannitol (pH 7.4, 310 mOsmol/kg-H_2_O).

### 4.3. Caspase Activity Measurements

Caspase activity was measured by using a fluorometric assay, as described previously [2,33]. To exclude an involvement of other proteases, the difference between fluorescence in the absence and presence of the specific inhibitor was observed. The fluorogenic substrates, which were labeled with fluorochrome 7-amino 4-methyl coumarin (AMC), for caspase-3 (Ac-DEVD-AMC), caspase-8 (Ac-IETD-AMC) and caspase-9 (Ac-LEHD-AMC) were provided from Promega (Madison, WI, USA), BIOMOL Research Labs (Plymouth, PA, USA) and Quality Controlled Biochemicals (Hopkinton, MA, USA), respectively. Specific inhibitors of caspase-3 (Ac-DEVD-CHO), casepase-8 (Ac-IETD-CHO) and caspase-9 (Ac-LEHD-CHO) were also obtained from these companies, respectively.

### 4.4. DNA Fragmentation Assay

Internucleosomal DNA fragmentation was detected by DNA ladder, as previously described [2,34]. The chromosomal DNA was analyzed by agarose gel electrophoresis (2%), followed by staining with ethidium bromide.

### 4.5. Cell Viability Assay

In the 96-well culture plates, cell viability was assessed by mitochondrial dehydrogenase activity using the colorimetric 3-[4,5-dimethylthiazol-2-yl]-2,5-diphenyltetrazolium bromide (MTT) assay [35], as described previously [2].

### 4.6. Chemicals

DIDS, phloretin, quinine, SITS and NPPB were purchased from Sigma and added to extracellular solution after solubilizing with a minimal amount of dimethylsulfoxide (DMSO). zVAD-fmk and zD-dcb were provided from Enzyme Systems Products (Livermore, CA, USA) and Peptide Institute (Osaka, Japan), respectively.

### 4.7. Statistical Analysis

Date given as means ± SEM of observations (n) were statistically analyzed using the ANOVA with Student’s *t* test. Differences were considered when *p* was <0.05

## 5. Conclusions

The present study was performed to address the question as to whether the early-phase caspase-3-independent apoptotic cell shrinkage, termed AVD, is dependent on the mitochondrial pathway and activation of initiator caspases. The AVD induction was observed by Fas-ligation in type-I SKW6.4 cells, which undergo Fas-mediated apoptosis without involving mitochondria. AVD was also observed in WEHI-231 cells treated with an intrinsic apoptosis inducer STS, even if the mitochondrial pathway is inhibited by overexpression of Bcl-2. Also, the AVD induction was found to precede activation not only of caspase-3, but also of caspases-8 and -9 in HeLa cells, and prevention of AVD induction by K^+^ and Cl^−^ channel blockers abolished activation of initiator and effector caspases. Furthermore, the AVD measurements in HeLa cells with broad-spectrum caspase inhibitors showed that the apoptotic whole-cell shrinkage comprises two phases: The early-phase caspase-independent AVD and the late-phase caspase-dependent persistent shrinkage. Thus, it is concluded that AVD is an early event independent of apoptotic mitochondrial dysfunction and activation of initiator and effector caspases, and that AVD is requisite to induce caspase activation and late-phase apoptotic cell shrinkage.
